# Effects of respiratory disease and age in quadriceps muscle mass: a pilot study with ultrasonography

**DOI:** 10.1080/07853890.2021.1897436

**Published:** 2021-09-28

**Authors:** Paula Martins, Laura Noronha, Alexandra André, Cátia Paixão, Patrícia Rebelo, Ana Oliveira, Silvia De Francesco, Alda Marques

**Affiliations:** a School of Health Sciences (ESSUA), University of Aveiro, Aveiro, Portugal; b Institute of Biomedicine (iBiMED), University of Aveiro, Aveiro, Portugal; c Institute of Electronics and Informatics Engineering of Aveiro (IEETA), University of Aveiro, Aveiro, Portugal; d Universidade Luterana do Brasil, Canoas, Brasil; e Respiratory Research and Rehabilitation Laboratory - Lab3R – School of Health Sciences, University of Aveiro, Aveiro, Portugal; f Coimbra School of Health, Polytechnic Institute of Coimbra, Coimbra, Portugal

## Abstract

**Introduction:**

Patients with chronic obstructive pulmonary disease (COPD) have been shown to present more muscle wasting than their healthy peers, which affects their quality of life [1,2]. The mechanisms behind muscle wasting in COPD are still little understood and even less is known in other chronic respiratory diseases (e.g. interstitial lung diseases – ILD) [3]. Ultrasound (US) is a safe and inexpensive imaging modality which can provide reliable measurements of muscle size and quality [2]. Thus, US may be a useful technique to enhance our understanding of muscle waste in chronic respiratory diseases. This study aimed to explore differences in quadriceps muscle mass in patients with COPD, hypersensitivity pneumonitis (ILD-HP) and healthy people (elderly and young).

**Materials and methods:**

A cross-sectional pilot study was conducted with 10 patients with ILD-HP (68.4 ± 9.8yrs), 10 patients with COPD (69.4 ± 6.7yrs) and 10 healthy elderly volunteers (67.8 ± 8.7yrs). Groups were gender (5f/5m) and age matched. A group of 10 young university students (21.9 ± 3yrs) was also included. An US equipment (GE LOGIQ P6) with multifrequency linear probe (10–13 MHz) was used to obtain B-mode US images. The following measures were taken: Rectus Femoris Thickness (RF_T_), Quadriceps Thickness (Q_T_) and Rectus Femoris cross sectional area (RF_CSA_). Data were analysed using SPSS version 24. Data normality and homogeneity were assessed. Between-group differences and correlations were performed with non-parametric tests (Kruskal–Wallis, Mann–Whitney *U*-test and Spearman´s correlation coefficient). Statistical significance was set at .05.

**Results:**

RF_CSA_ (median and IQR) was 5.44 [3.56–6.57] cm^2^; 4.29 [3.58–4.50] cm^2^; 6.06 [4.61–9.41] cm^2^ and 7.99 [5.92–9.41] cm^2^ for ILD-HP, COPD, elderly and young people, respectively. RF_T_ results were 1.51 [1.08–1.78] cm; 1.16 [1.07–1.53] cm; 1.64 [1.36–1.76] cm and 2.06 [1.68–2.27] cm, respectively. There were significant differences in RF_CSA_ (*p* = .027), RF_T_ (*p* = .041) and Q_T_ (*p* = .011) between COPD and elderly people. No differences were found between ILD-HP group and elderly. Significant differences between the elderly and young groups were found for the same measurements (RF_CSA_ *p* = .034; RF_T_ *p* = .016; Q_T_ *p* = .034). Moderate and negative correlations were found between age and RF_CSA_ (*r*_s_=–0.416), RF_T_ (*r*_s_=–0.540) and Q_T_ (*r*_s_=–0.450). A strong and positive correlation was found between RF_T_ and RF_CSA_ (*r*_s_=0.891).

**Discussion and conclusions:**

Our results seem to corroborate previous findings supporting the existence of quadriceps muscular wasting in patients with COPD when compared with age-matched healthy controls [2,4]. In the group of patients with ILD-HP, muscle mass seems to be somewhat preserved. To confirm our results, future studies should include a larger sample with quantitative measures of muscular quality (e.g. echointensity) and relationship between muscle size/quality and muscle strength.

## References

[1] Seymour JM, Ward K, Sidhu PS, et al. Ultrasound measurement of rectus femoris cross-sectional area and the relationship with quadriceps strength in COPD. Thorax. 2009;64(5):418–423. 19158125 10.1136/thx.2008.103986

[2] Menon MK, Houchen L, Harrison S, et al. Ultrasound assessment of lower limb muscle mass in response to resistance training in COPD. Respir Res. 2012;13(1):119. 23273255 10.1186/1465-9921-13-119 PMC3560243

[3] Mendes P, Wickerson L, Helm D, et al. Skeletal muscle atrophy in advanced interstitial lung disease. Respirology. 2015;20(6):953–959. 26081374 10.1111/resp.12571

[4] Shrikrishna D, Patel M, Tanner RJ, et al. Quadriceps wasting and physical inactivity in patients with COPD. Eur Respir J. 2012;40(5):1115–1122. 22362854 10.1183/09031936.00170111

